# Comparison of human papillomavirus detection between freshly frozen tissue and paraffin embedded tissue of invasive cervical cancer

**DOI:** 10.1186/1750-9378-5-15

**Published:** 2010-09-16

**Authors:** Michael Odida, Silvia de Sanjose, Sven Sandin, Beatriz Quiros, Laia Alemany, Belen Lloveras, Wim Quint, Bernhard Kleter, Maria Alejo, Leen-Jan van Doorn, Elisabete Weiderpass

**Affiliations:** 1 Department of Medical Epidemiology and Biostatistics, Karolinska Institutet, Stockholm, Sweden; 2 Department of Pathology, Faculty of Medicine, Makerere University, Kampala, Uganda; 3 Unit of Infections and Cancer, Cancer Epidemiology Research Programme (IDIBELL), Institut Català d' Oncologia, Barcelona, Spain; 4 The Biomedical Research Centre Network for Epidemiology and Public Health (CIBERSP), Barcelona, Spain; 5 Department of Pathology, Hospital del Mar, Barcelona, Spain; 6 DDL Diagnostic Laboratory, Voorburg, The Netherlands; 7 Department of Pathology, Hospital General de Vic, Barcelona, Spain; 8 Department of Etiological Research, Cancer Registry of Norway, Oslo, Norway; 9 Department of Community Medicine, Tromso University, Tromso, Norway; 10 Department of Genetic Epidemiology, Folkhälsan Research Center, Helsinki, Finland

## Abstract

**Background:**

Human Papillomavirus (HPV) detection results comparing paraffin embedded cervical tissue and other cervical specimens have been done with varying degrees of agreement. However, studies comparing freshly frozen specimens and paraffin embedded specimens of invasive cervical carcinomas are lacking. The aim of the study was to compare HPV detection using SPF_10 _broad-spectrum primers PCR followed by DEIA and genotyping by LiPA_25 _(version 1) between freshly frozen cervical tissue samples and paraffin embedded blocks of cervical tissue from the same patient. There were 171 pairs of paraffin embedded and freshly frozen samples analyzed from cervical carcinoma cases from Kampala, Uganda.

**Results:**

88.9% (95% CI: 83.2%-93.2%) of paraffin embedded samples were HPV positive compared with 90.1% (95% CI: 84.6%-94.1%) of freshly frozen samples, giving an overall agreement in HPV detection between fresh tissue and paraffin embedded tissue at 86.0% (95% CI: 79.8%-90.8%). Although the proportion of HPV positive cases in freshly frozen tissue was higher than those in paraffin blocks, the difference was not statistically significant (p > 0.05). In both types of tissues, single HPV infections were predominant, with HPV16 accounting for 47% of positive cases. Comparison in the overall agreement, taking into accounts not only positivity in general, but also HPV types, showed a 65% agreement (complete agreement of 59.7%, partial agreement of 5.3%) and complete disagreement of 35.0%. HPV detection in squamous cell carcinomas (SCC) and adenocarcinomas (ADC) was similar in fresh tissue or paraffin blocks (p ≥ 0.05).

p16 immunostaining in samples that had at least one HPV negative results showed that 24 out of 25 cases had an over-expressed pattern.

**Conclusions:**

HPV DNA detection was lower among ADC as compared to SCC. However, such differences were minimized when additional p16 testing was added, suggesting that the technical issues may largely explain the HPV negative cases.

## Background

Improved DNA retrieval methods have made possible the realization of gene analyses and viral genome identification in archival formalin fixed paraffin embedded (FFPE) tissues [1-4]. A number of methods have been developed over the past few years to detect the Human Papillomavirus (HPV), one of them being Short PCR Fragment-10 (SPF_10_) Line Probe Assay system (LiPA) [5,6]. This assay is based on the amplification of a 65-bp region of L1 open reading frame. In order to validate the SPF_10 _LiPA assay using FFPE cervical samples, comparisons with other samples have been performed by some investigators [7,8] with good overall agreement. The overall agreement was generally high from these studies, when cervical scrapes or cytological samples were used. Besides the generally lower detection of HPV in FFPE samples of cervical carcinoma, it has been shown that adenocarcinomas show an even lower positive rate compared to other histological types [9]. In view of these discrepancies, we thought it worthwhile to conduct a study to clarify whether the formalin fixation could differentially affect HPV detection according to the histological diagnosis. The main objective of the present study was to compare HPV detection using SPF_10 _broad-spectrum primers Polymerase Chain Reaction (PCR) followed by Deoxyribonucleic Acid Enzyme Immunoassay (DEIA) and genotyping by LiPA_25 _(version 1) between fresh cervical tissue samples and paraffin embedded blocks from the same patients, and also to assess whether there are differences in HPV detection between squamous cell carcinomas and adenocarcinomas.

## Methods

The materials for this study were obtained as part of a case-control study  conducted at Mulago Hospital, Kampala, Uganda which is the teaching hospital for the College of Health Sciences of Makerere University from September 2004 to December 2006. Tumour specimens were obtained from new incident cases of cervical carcinoma presented to the hospital as part of routine care. The tumour specimens were divided into two parts. One part was fixed in 10% formalin and entered in the register of Department of Pathology for processing (paraffin blocks), while the other part was stored at -80°C until transferred to the laboratory for analysis. In cases where the tissue was deemed to be very small, the whole tissue was formalin fixed and processed for histopathological diagnosis. Each case was given an identification number so that the paraffin block could be linked with the fresh specimen. The samples from the cases were all biopsies. The local histopathological diagnosis was performed by Michael Odida.

### HPV detection of cervical cancer cases in freshly frozen tissue

HPV detection and genotyping in 195 freshly frozen cervical cancer specimens was performed at the DDL Diagnostic Laboratory in The Netherlands during the period from June to August 2008, also 1,5 up to 4 years after diagnosis. HPV detection was performed using SPF_10 _broad-spectrum primers PCR followed by DEIA and genotyping by LiPA_25 _(version 1).

### HPV detection of cervical cancer cases preserved in paraffin

The paraffin embedded blocks were prepared in Uganda for diagnostics purposes between September 2004 and December 2006 when the patients were recruited for the study. HPV detection and genotyping of 201 paraffin blocks was performed by pathologists at the Unit of Infections and Cancer at the Catalan Institute of Oncology (ICO) during January 2010, also 3 to 5.5 years after diagnosis. The detailed methods have been previously described in Odida et al. [10]. Briefly, at ICO the samples were processed following the next steps: (a) Re-embedding of the tissue material was done if necessary when the paraffin block was in poor condition for cutting; (b) Microtome sectioning of the specimens under non-contamination conditions and the sandwich technique were carried out to confirm an optimal number of sections to be used for DNA extraction and testing; (c) All cases were reviewed by a trained pathologist at ICO for diagnosis and assessing quality of the specimen before HPV testing. Cases difficult to classify, cases with a discordant diagnosis compared to the field diagnosis and all the rare histological types were further reviewed by two senior expert pathologists at ICO; (d) DNA was extracted under non-contamination protocols and aliquoted, and HPV testing was performed on each specimen using the SPF-10 broad spectrum primers PCR followed by DEIA. HPV DNA positive samples were subsequently analyzed by LiPA_25 _(version 1: produced at Laboratory Biomedical Products, Rijswijk, The Netherlands), a reverse hybridization technique that detects 25 high-risk (HR) and low-risk (LR) HPV types (6, 11, 16, 18, 31, 33, 34, 35, 39, 40, 42, 43, 44, 45, 51, 52, 53, 54, 56, 58, 59, 66, 68, 70, 74). Both labs, DDL and ICO, performed routine cross-validation testing for SPF_10_, DEIA and LiPA with high agreement between them, concordant results of >98% (data not shown).

Since the number of samples received at DDL and ICO differ, the comparison was performed based on the cases that were HPV analyzed in both laboratories, n = 171. Data generated at Makerere Hospital (original pathological diagnosis), at DDL (HPV detection in freshly frozen tissue) and at ICO (pathological diagnosis and HPV detection in paraffin blocks) were merged into one dataset using the common identifier first provided by Makerere University. A descriptive analysis of global HPV positivity and HPV types globally and by histological diagnosis of cervical carcinoma and type of specimens was performed. Global HPV detection concordance analysis, in terms of positive or negative result was done to assess differences between the fresh and paraffin, as well as between squamous cell carcinoma (SCC) and adenocarcinomas (ADC).

Further analysis for the overall agreement, taking into account not only general positivity but also HPV types, was done to assess the exact agreement between the two samples. The agreement was classified as complete, partial or complete disagreement according to the definitions below.

#### Complete agreement

The result is exactly the same (same HPV single type, or same multiple types or HPV negative result).

#### Partial agreement

The cases have at least one identical type.

#### Complete disagreement

The cases are totally different (different type or HPV positive-HPV negative).

### p16 Immunohistochemistry

It has been largely described that p16 over-expression is a surrogate marker of HPV E7 oncoprotein-mediated catabolism of pRb in premalignant and malignant lesions of the cervical mucosa. In the present study, p16 immunostaining was performed in a selected number of paraffin embedded tissue from cancer cases in order (1) to obtain more insight regarding the HPV oncogenic role in the discordant cases and (2) to discard non-HPV related cancers in the concordant HPV negative cases. The cases included in this analysis were all HPV discordant cases (n = 24), all concordant HPV negative cases (n = 6) and a random sample of approximately a 10% of the concordant HPV positive cases (n = 15). p16INK4a was detected using the CINtec histology kit (clone E6H4, MTM Laboratories, Heidelberg, Germany), following the manufacturer's protocol. In each series, a negative control, and a positive control consisting of an invasive cervical carcinoma were included. The percentage of stained cells (-: < 5%; +: 5-25%; ++: 26-75%; +++: > 75%) and the pattern (focal/diffuse) was recorded, and a positive case was considered when the percentage was 5 and above (++ or more) with a diffuse pattern.

### Statistical tests

The Kappa statistics and McNemar test were used to test the statistical significance of HPV detection between fresh samples and paraffin blocks, while the Chi square test and Fisher exact test were used to test the statistical significance of HPV detection between SCC and ADC in both fresh and paraffin blocks. Where appropriate, 95% confidence intervals (CI) were computed, and the level of statistical significant test was set at 0.05.

## Results

### Overall agreement between fresh tissue specimens and paraffin embedded tissue

Overall, 154 of freshly frozen tissue were positive for HPV (90.1%, 95% CI: 84.6%-94.1%) and 152 of tissue in paraffin blocks were HPV positive (88.9%, 95% CI: 83.2%-93.2%), giving an overall agreement in HPV detection between fresh tissue and paraffin blocks at 86.0% (95% CI: 79.8%-90.8%), which was statistically significant (Kappa Index = 0.26, p = 0.001). Although the proportion of HPV positive cases in fresh tissue was higher than those in paraffin blocks, the difference was not statistically significant (McNemar test, p > 0.05). The details are shown in Table 1.

**Table 1:** HPV detection in fresh tissue versus paraffin block

		Paraffin block				Total	
				
		Positive		Negative			
		
		N	**%**^**a**^	N	**%**^**a**^	N	**%**^**a**^
Fresh tissue	Positive	141	82.5^b^	13	7.6	154	90.1
	Negative	11	6.4	6	3.5^b^	17	9.9
	Total	152	88.9	19	11.1	171	100

^a ^Percentage of cell number among all the cases (171)

^b ^Concordant results

Concordance analysis-Kappa Index: value of the index 0.26; p = 0.001

McNemar test: p > 0.05

In both types of tissues, single HPV infections were predominant. Only one case has been DEIA positive, and LiPA negative (HPVX, unknown type) and has been detected in paraffin embedded tissue. The proportion of multiple HPV infections was higher in freshly frozen tissue cases than tissue in paraffin blocks. However, the differences between both kinds of tissues were not statically significant (all p-values are > 0.05). The details are shown in Table 2.

**Table 2:** HPV detection in fresh tissue versus paraffin block: single/multiple/HPVX types

HPV detection	Fresh tissue			Paraffin block		
	
	Number of cases	%	**95% CI**^**b**^	Number of cases	%	**95% CI**^**b**^
Cases HPV analyzed	171	100		171	100	
HPV negative	17	9.9	(5.9-15.4)	19	11.1	(6.8-16.8)
HPV positive	154	90.1	(84.6-94.1)	152	88.9	(83.2-93.2)
Single HPV types^a^	143	92.9	(87.6-96.4)	144	94.7	(89.9-97.7)
Multiple HPV types^a^	11	7.1	(3.6-12.4)	7	4.6	(1.9-9.3)
HPV X - unknown^a^	0	0.0	(0.0-2.4)	1	0.7	(0.0-3.6)

CI, Confidence Interval;

^a ^Number of single/multiple/X among HPV positive cases;

^b ^For all comparisons: p-value > 0.05

### HPV types in single infections

**Figure 1:**
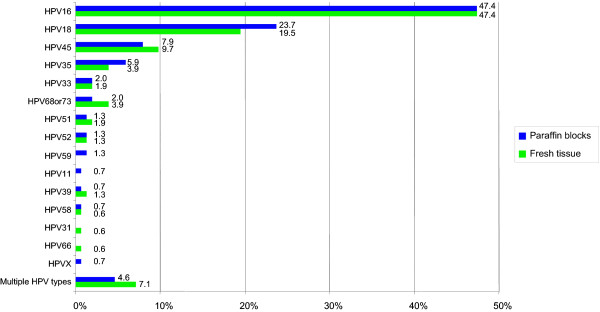
**HPV type distribution of single types in fresh tissue versus paraffin block**. Percentiles (%) of single/multiple/X HPV types among HPV positive cases

In both kinds of tissue, HPV16 was the most frequently identified in 47.4% among HPV positive cases. The proportion of HPV33, HPV52 and HPV58 were almost identical. A higher proportion of HPV18 and HPV35 were identified in paraffin blocks compared to fresh tissue and, conversely, a higher proportion of HPV45, HPV68 or 73, HPV51 and HPV39 in fresh tissue. HPVs 66 and 31 were only identified in fresh tissue and HPVs 59, 11 and HPVX were only detected in paraffin embedded tissue. The details are shown in Figure 1.

### HPV types in multiple infections

Table 3 shows HPV type distribution of multiple types in freshly frozen tissue versus tissue in paraffin blocks. In 11 out of the 16 cases described in table 4, the HPV result matched at least for one of the HPV types.

**Table 3:** HPV type distribution of multiple types in fresh tissue versus paraffin block

**Fresh tissue**^**a**^	**Paraffin block**^**b**^
HPV **18 & 74**	HPV **18 & 74**
HPV **16 & 52**	HPV **16 & 52**
HPV **16**	HPV **16 **& 35
HPV **18**	HPV 16 &**18**
HPV **18**	HPV **18 **& 56
HPV **45**	HPV **45 **& 54
HPV **16 **& 45	HPV **16**
HPV **18 **& 31	HPV **18**
HPV **18 **& 70	HPV **18**
HPV **59 **& 74	HPV **59**
HPV **39**	HPV 16 &**39**
HPV 16 & 18	HPV 59
HPV 18 & 52	HPV 16
HPV 6 & 44 & 45	HPV 18
HPV 39 & 51 & 52	HPV 33
HPV 6 & 45	HPV negative

^a ^Multiple HPV cases, N = 11

^b ^Multiple HPV cases, N = 7

**Table 4:** HPV detection in fresh tissue versus paraffin block according to histological diagnosis

HPV detection	SCC N = 130			ADC N = 7		
	
	Number of cases	%	95% CI	Number of cases	%	95% CI
Fresh tissue	119	91.5	(85.4-95.7)	6	85.7	(42.1-99.6)
Paraffin blocks	121	93.1	(87.3-96.8)	5	71.4	(29.0-96.3)

ADC, adenocarcinoma; CI, confidence interval; SCC, squamous cell carcinoma;

For all comparisons: p-value > 0.05

### HPV types agreement between fresh tissue specimens and paraffin embedded specimens

When analyzed for the overall agreement, also taking into account HPV types, complete agreement was found to be 59.7% (complete agreement in single HPV types: 55.0%, complete agreement in multiple types: 1.2%, complete agreement in HPV negativity: 3.5%), partial agreement: 5.3% and complete disagreement: 35.0%.

### HPV detection concordance by histological type of cervical carcinoma

For performing the HPV detection concordance analysis stratified by histological diagnosis, we selected the cases that had a concordant histological diagnosis between the local and ICO pathology evaluation: SCC (n = 130) and ADC (n = 7).

### Sqamous cell carcinoma

The HPV positivity in freshly frozen tissue was 91.5% (95% CI: 85.4%-95.7%), and the corresponding HPV positivity in paraffin embedded tissue was 93.1% (95% CI: 87.3%-96.8%), giving an overall agreement in HPV detection between fresh tissue and paraffin embedded tissue of 90.8% (95% CI: 84.4%-95.1%), which was statistically significant, although the strength of concordance is weak (Kappa Index of agreement of 0.35; p < 0.001). Although identifying a higher HPV positivity in tissue from paraffin blocks, this difference was not statistically significant (McNemar test, p > 0.05).

### Adenocarcinoma

The HPV positivity in freshly frozen tissue was 85.7% (95% CI: 42.1%-99.6%), and the HPV positivity in paraffin embedded tissue was 71.4% (95% CI: 29.0%-96.3%), giving an overall agreement in HPV detection between fresh tissue and paraffin blocks: 85.7% (95% CI: 42.1%-99.6%), which was not statistically significant (Kappa Index of agreement of 0.59; p > 0.05). Although identifying a higher HPV positivity in fresh tissue, this difference was not statistically significant (McNemar test, p > 0.05).

### HPV detection concordance between histological types of cervical carcinoma

Comparing HPV positivity by diagnosis and tissue preservation, adenocarcinomas were more likely to be HPV negative than SCC, both in freshly frozen tissue and paraffin embedded tissue. This difference was higher in paraffin embedded tissue than in fresh tissue (21 percentage points difference versus 6, respectively), although the differences were not statistically significant (p > 0.05), as is shown in Table 4.

### p16 Immunostaining results

45 cases were included in the p16 analysis. From these cases p16 was successfully performed in 40 (88.9%). In the other 5 cases, there was no tumour in the p16 slide (11.1%). All cases except one showed a positive p16 over-expressed pattern (Table 5). Only one case, an adenocarcinoma NOS (subtype: not other specified), was HPV negative in both fresh and paraffin embedded tissue and also showed a p16 negative pattern, suggesting that maybe it is not HPV related or it is a case with an endometrial origin (not cervical).

**Table 5:** Results of p16 expression by HPV results

HPV results fresh/paraffin preservation	p16							
			
	-		++		+++		Total	
	
	N	%	N	%	N	%	N	%
Concordant HPV negative	1^a^	16.7	1^b^	16.7	4^e^	66.7	6	100
Concordant HPV positive	0	0.0	1^c^	6.7	14^f^	93.3	15	100
Fresh HPV positive, Paraffin HPV negative	0	0.0	0	0.0	9^g^	100	9	100
Fresh HPV negative, Paraffin HPV positive	0	0.0	2^d^	20	8^h^	80	10	100

Total	1	2.5	4	10	35	87.5	40	100

p16-: < 5% stained cells; p16++: 26-75%; p16+++: > 75%

Histologies:

^a ^adenocarcinoma NOS (not other specified)

^b ^undifferentiated carcinoma

^c ^undifferentiated carcinoma

^d ^1 adenocaricnoma and 1 undifferentiated carcinoma

^e ^4 squamous cell carcinoma

^f ^13 squamous cell carcinoma and 1 undifferentiated carcinoma

^g ^5 squamous cell carcinoma, 1 adenocarcinoma and 3 undifferentiated carcinoma

^h ^7 squamous cell carcinoma, 1 adenocarcinoma

## Discussion

The goal of this study was to examine whether HPV genotyping on FFPE invasive cervical carcinoma specimens give comparable results with freshly frozen specimens obtained from the same patient simultaneously. Our study suggests that detection of HPV using SPF_10 _with LiPA technology using formalin fixed paraffin embedded tissues gives comparable results to freshly frozen specimens for HPV detection in general (Kappa index 0.26, p = 0.001), see Table 1, but is not so good for specific HPV types detection (p-value > 0.05). Some previous studies which compared the paraffin embedded tissue with other samples found high agreement [7,8,11]. However, these studies used exfoliated cells or cervical scrapes and not freshly frozen specimens. Besides, these were cases of preneoplastic lesions, not invasive cervical carcinoma. A study which compared FFPE tissue with cervical scrapes from women with invasive cervical carcinoma found very high HPV type specific concordance [12]. However, the number of samples studied was small, and also the method of detection was GP5+/6+ primers.

Another interesting observation was the negative HPV results in both freshly frozen specimens and FFPE tissues. Our analysis of p16 immunostaining in samples with at least one negative result showed that all but one sample tested p16 positive, suggesting that the cases are HPV mediated and that the most probable reason for not detecting the virus is related to viral, specimen preservation or technical issues such as denatured DNA by alkali [13] or by formalin fixation [14]. For specific HPV types, if one considers fresh specimens as gold standard, then discordance in HPV results from FFPE specimens could be due to a number of explanations. One is that formalin fixation could have denatured the tissue. It is known that DNA extracted from FFPE tissues are usually at low concentration and fragmented [14,15]. Another alternative view is reduced sensitivity detection of some HPV types in paraffin specimens, especially types 42, 16, 18, 39, 56, 58, 59 and 66 [16-18]. A third possibility, which may be applicable to discordance in multiple infections is due to competition between different HPV genotypes [19]. It is conceivable that lower discordance rates could be obtained by reducing the duration of fixation in buffered formalin.

The low detection of HPV in adenocarcinoma in both types of specimens is also of interest. A number of previous studies have also found low detection of HPV in adenocarcinoma of the cervix [9,20,21]. Plausible explanations are: (a) that some cervical tumours may be of endometrial origin and are therefore HPV negative [22]; (b) that some cervical adenocarcinomas may not be related to HPV (such as minimal deviation adenocarcinomas) [21,23]; (c) low viral load; (d) fewer episomal copies; (e) or loss of viral genome during integration in cervical adenocarcinoma [24]. Though some investigators found a high positive rate which they attributed to higher diagnostic accuracy and exclusion of non cervical tumours [25].

Our results have some implications. For areas with low resources, use of FFPE samples for HPV detection is feasible, especially HPV testing for clinical management of women with abnormal smears. In case a biopsy is taken, the paraffin embedded tissue could be referred to laboratory for HPV testing. This would make use of HPV results, whether for prognosis or detection of progression of cervical lesions like high-grade squamous intraepithelial lesion (HSIL) possible in many areas. However, there would be a need to have standard protocols so that fixation time is controlled. In some situations, the preference for fresh or paraffin tissue will depend on the amount of available tissue and the questions being addressed.

Our study had some strengths. Compared with a recent study which compared the use of cervical scrapes and tissues from women with cervical carcinoma [12], our sample size was big. The specimens were analyzed in two different laboratories blindly using the same HPV detection method. The SPF_10 _and LiPA technology are quite sensitive and specific for HPV [16]. The study also has, however, some limitations. The piece of tissue used was divided in two, and half was used to prepare the paraffin blocks and half kept as freshly frozen tissue for HPV genotyping. Thus, they were not exactly the same tumour materials, but pieces of tissue adjacent to each other. Moreover, a small number of adenocarcinomas limits the use of statistical tests.

## Conclusions

There were no differences in HPV positivity between freshly frozen tissue and paraffin embedded tissue in general, but for specific types, only 65% of the cases had complete or partial agreement on the HPV detection. HPV DNA detection was lower in ADC as compared to SCC, the difference being higher in paraffin blocks than in fresh tissue (21 percentage points difference versus 6, respectively; p > 0.05). However, such differences were minimized when additional p16 testing was added, suggesting that technical issues largely explain the HPV negative cases.

## List of abbreviations

ADC: adenocarcinoma; CI: confidence interval; DDL: Diagnostic Laboratory; DEIA: deoxyribonucleic acid enzyme immunoassay; FFPE: formalin fixed paraffin embedded; HPV: human papillomavirus; HSIL: high-grade squamous intraepithelial lesions; ICO: Catalan Institute of Oncology; INK4: tumour suppressor protein; LiPA: line probe assay; p16INK4a: prototypic INK4 protein on p16 gen, identified as a tumour suppressor in many human cancers; PCR: polymerase chain reaction; SCC: squamous call carcinoma; SPF_10_: short PCR fragment.

## Competing interests

The authors declare that they have no competing interests.

## Authors' contributions

MO, EW and SdS were responsible for developing concept, full proposal development and getting ethical approvals. MO was responsible for samples selection, histological review of origin and data cleaning. SS, BQ and LA were responsible for data analysis. BL and MA were responsible for histological review of the paraffin cases and p16 analysis. WQ, BK and LJvD were responsible for HPV DNA detection in fresh samples. MO, EW and SdS wrote the manuscript. All co-authors: SS, BL, BQ, LA, WQ, BK, LJvD, MA revised and gave valuable comments to the first draft of this manuscript. MO and EW were responsible for preparing the manuscript for submission. All authors approved the final version submitted.
